# Textile Supplies for Hospitals

**Published:** 1916-04-01

**Authors:** 


### TEXTILE SUPPLIES FOR HOSPITALS.

### VIII. Sheets and Quilts.

Measure for measure, sheets are not the most expensive fabrics in hospital use, but their number makes them the principal item of expense in equipping beds, and the expenditure upon them is recurrent. Although they are articles of an eminently simple type, marked differences are evident between the wearable properties of separate qualities, and some different ideas as to the comparative values of different kinds obtain. Two main varieties exist—linen sheets and cotton ones—both made in various qualities. Linen sheets are for natural reasons the more expensive, and when their cost does not exceed that of cotton by more than a certain margin they are the more economical article to buy. The ratio of cost between the two sorts is not constant, for flax and cotton are separate commodities, subject to independent influences, and a European war, for example, by curtailing the supplies of flax, drives up the price of linen, although cotton may for a time actually cheapen. Good linen sheets are certainly stronger than cotton ones, although their elasticity is lower, and usually they bear a larger number of launderings. Linen is generally smoother to the touch, and on first contact it feels perceptibly colder to the skin. In some circles and some parts of the country where linen sheets are associated distinctively with the dead, their use excites an aversion which must be reckoned with in homes for the sick.

### Bleached and Unbleached.

Cotton sheets are the more largely used and are produced upon a much greater scale than linen ones, and in devoting attention chiefly to them it may be added that what applies to the one applies in large part also to the other. In choosing between sheets of either sort, the question of bleached or unbleached arises. A previous article in this series has dealt with the nature and extent of the protoplasmic matter present upon the natural or unbleached fibre. The colouring matter and the waxes which bind it to the fibre are gradually removed by successive washings after unbleached sheets have been bought. The articles grow whiter in use, especially if they are dried in sunshine, and gradually they lose weight, although no records of this loss are ordinarily taken. An unbleached cotton or linen sheet is stiffer, darker, and heavier than the same article bleached; in short, there is more of it, and these superficial appearances may well have an influence in causing unbleached goods to be preferred, and all the more because bleaching costs money and adds to the price.

Bleaching manifestly removes part of the substance of the fabric, and this fact, coupled with some prejudice against the means by which commercial bleaching is done, perhaps assists the preference for the unbleached article. The processes of bleaching vary with the conditions of the case, but they are severe in any event. The cloth—or it may be the yarn—is boiled in high-pressure chambers successively in a quicklime solution and in alkali. These processes for loosening the waxy matters are followed by a de-colouring treatment in a chlorine bath. Twice in the course of operations the material is treated in dilute acids, and it is most thoroughly washed to eliminate traces of chemicals which would, if left undisturbed, rot the fibre. The operations sound destructive, and in point of fact bad bleaching does diminish the strength as well as the weight of the material. On the other hand, good bleaching demonstrably leaves the fabric stronger, despite that some of its constituents are removed. The severe processes induce changes within the fibre itself, and in some conditions the gain is highly considerable; gains of as much as 60 per cent. in strength have been noted, although a proportion more like 10 per cent. is usual upon tightly twisted cotton yarn. Bleached sheets are preferable from the point of view of appearance, and it is of some importance to know that bleaching does not involve loss of strength.

### Twilled or Plain.

Opinion is divided as to the respective advantages of buying twilled sheets or plain ones, and it is significant that the difference only arises in respect of cottons. Linen sheets are plain woven, as a matter of course, and in cotton buyers are offered their choice. In plain weaving the threads are crossed one-over one-under alternately, and in twill are so arranged that the point of crossing forms a diagonal rib. Twills are of innumerable constructions, the most elementary being that in which the threads run two-over and two-under, with a shift of the alternation of threads at every successive course of the weft. There is no better bound or better balanced fabric than one made with a plain weave, and to that extent plain sheeting should be best in wearing power. The cloth is, however, made more bulky by using the twill weave, and the play of light along the rib assists the cloth to show to good advantage. The smarter appearance and fuller " handle " of the twill foster the impression that the sheets come up better after washing than the flat plains. The reasoning does not, however, explain the opinion, prevalent in some directions, that twill sheets last better in wear. The case is probably not so where identical yarns are used in each instance. It has been already said that stronger and more closely-twisted yarns are commonly used for warp than for weft. In a sheet woven plain from threads of equal thickness, equal quantities of warp and weft are present upon the surface; but in a twill the arrangement can be made to throw a preponderance of the stronger threads on the upper surface. The truth may well be that some twill sheetings wear better than some plains, although the latter can be made, by use of twist yarn in the weft, to wear better than the former.

Note.—Previous articles in this series appeared on December 18, 1915, and January 8 and 22, February 5 and 19, and March 4 and 18, 1916.

### Fictitious Weight.

The cheapest cotton sheets have a relatively strong warp and a flimsy weft made from waste-cotton, with a plentiful addition of dressing or sizing. The size comes away in the wash-tub, which fills with the slime of starches, mineral and other ingredients used to add weight and firmness to intrinsically poor material. Sheetings made from cotton-waste are commonly thicker, and have a more lumpy and irregular rib than twills made from good cotton. Dressing or sizing is a necessity to good weaving, and so long as it is kept within the limits required strictly for the purposes of weaving it is unobjectionable. The necessary amount is usually less than 10 per cent., but sheets are sometimes made with a loading of 30 per cent., and there are cottons that are weighted 100 per cent. and more with mixtures of flour, clay, tallow, and magnesium and zinc chlorides.

The two ways of securing the best value in sheets are to buy only makes known for their high and consistent quality, which are made by several manufacturers; or, alternatively, to buy upon a standard specification. A specification used by a large company of lodging-house proprietors is given below, and may serve as a model:—

Specification for Unbleached (Plain Cotton Sheeting.)

Width.—54 inches.

Weight per lineal yard.—11¾ to 12 oz.

Threads per inch warp.—60.
Threads per inch weft.—60.

Strength 9 in. × 4 in. warp.—360 lb.

9 in. between jaws weft.—300 lb.

Foreign matter, size, etc.—Not exceeding 10 per cent.

*Note.*—To show style and make a portion of the standard pattern is attached hereto. Details of the standard specification must, however, in all cases be adhered to. The sealed standard may be seen by appointment at........................................

The stated weight per lineal yard increases proportionately with the width of the cloth, which, in the specification, is somewhat narrow for hospital use. The threads per inch in relation to the weight fix the fineness of the yarn to be employed; and the breaking strength, here given for strips of 9 × 4 inches, serves to safeguard the quality. There could be no difficulty in obtaining sheets of the same dimensions and indistinguishable in appearance at less than the price for materials equal to the stated tests, but they are less suitable for the work. The breaking strains mentioned represent some 12½ to 15 per cent. less than the average normally recorded in sheetings of proven suitability, and the limits suffice to keep up the quality of the sheets purchased without burdening the manufacturer with unreasonable demands. The specification is not recommended for universal use, but is given as a specimen of the requirements that large consumers may with advantage have framed for themselves. In the case in point the same fabric that is used for sheeting is employed for pillows and bolsters, with the effect that considerable orders can be given for cloth of one quality. Standard specifications are more immediately useful to large institutions than small ones, and most so to those which aim at buying directly from the manufacturer. It has always to be reflected that the order which looks large in the hospital scale of proportions, looks less to the manufacturer making sheeting by the mile, but orders for special qualities, deserving of quite respectful attention, can be given out by institutions of 700 to 1,000 beds.

### Cotton Quilts.

Quilts, the cotton concomitants of sheets, have a manifestly different office, exposing them to less friction in wear, and calling for less frequent washing. The quilt is in effect an outer blanket supplying a modicum of warmth and selected, at least partly, for the sake of its appearance. In conventional quilts there is a certain width of choice between plain and coloured and smooth and honeycombed. The colouring is a matter of taste, and in selecting quilts decorated with colours precautions are advisable against colours which fade or "bleed." The cheapest dyes used for cottons have little to recommend them in those respects, and the so-called common colours, being those which blacken at a touch with acid, are to be avoided. The most satisfactory colour for long service is dull red, and for the reason that alizarin red, the coal-tar equivalent of the madder dye, is the colouring agent. If blue be preferred for æsthetic reasons, an indigo blue is to be recommended.

Since they have not to bear heavy strains quilts are articles in which short cotton fibre may advisably be used for economy, and a great many of them are made with a weft of waste-cotton. The better the quality of the quilt, the more even and less spongy are its yarns; and for hospital purposes the preference must be awarded rather to the closely twisted and woven articles which make for cleanliness and long wear, than to the looser texture which may have an advantage in respect of warmth. In a specification in current use the particulars are as follows:—

Specification for Quilts.

Length.—90 inches before hemming.

Weight.—3 to 3¼ lb. per quilt.

Foreign matter.—Not exceeding 3 per cent.

Dye.—The blue threads to be pure indigo.

Remarks.—The following lettering to be woven in 2-inch letters on a 4-inch collar in the centre of the quilt.

In ordering a certain quantity of quilts no difficulty is raised about weaving into them a name or monogram. Whether the device is in white or in coloured threads the means is one and the same. The article is woven on the Jacquard loom, and by an elaborate arrangement of cords threads are selected from the rest to rise or fall in accordance with the setting of cards punctured with circular holes. Blank cards have to be punched specially for each device, but once these have been obtained, the weaving of a name or monogram presents no more difficulty than weaving any floral or geometrical pattern.

[The next article in the series will deal with "Coloured Cottons for Uniforms."]

